# Prediction of the potentially suitable areas of *Leonurus japonicus* in China based on future climate change using the optimized MaxEnt model

**DOI:** 10.1002/ece3.10597

**Published:** 2023-10-19

**Authors:** Yongji Wang, Liyuan Xie, Xueyong Zhou, Renfei Chen, Guanghua Zhao, Fenguo Zhang

**Affiliations:** ^1^ School of Life Science Shanxi Normal University Taiyuan China

**Keywords:** climate change, ENMeval, *Leonurus japonicus*, MaxEnt, niche modeling

## Abstract

*Leonurus japonicus* Houtt. is a traditional Chinese medicinal plant with high medicinal and edible value. Wild *L. japonicus* resources have reduced dramatically in recent years. This study predicted the response of distribution range of *L. japonicus* to climate change in China, which provided scientific basis for the conservation and utilization. In this study, 489 occurrence points of *L. japonicus* were selected based on GIS technology and spThin package. The default parameters of MaxEnt model were adjusted by using ENMeva1 package of R environment, and the optimized MaxEnt model was used to analyze the distribution of *L. japonicus*. When the feature combination in the model parameters is hing and the regularization multiplier is 1.5, the MaxEnt model has a higher degree of optimization. With the AUC of 0.830, our model showed a good predictive performance. The results showed that *L. japonicus* were widely distributed in the current period. The maximum temperature of warmest month, the min temperature of coldest month, the precipitation of wettest month, the precipitation of driest month, and altitude were the main environmental factors affecting the distribution of *L. japonicus*. Under the three climate change scenarios, the suitable distribution area of *L. japonicus* will range shift to high latitudes, indicating that the distribution of *L. japonicus* has a strong response to climate change. The regional change rate is the lowest under the SSP126‐2090s scenario and the highest under the SSP585‐2090s scenario.

## Graphical abstract

The MaxEnt model with optimized parameters was used to predict the suitable habitats of *L. japonicus* at different stages, and the Marxan model was used to determine the priority of protected areas. The extremes of heat and cold, the precipitation in the wettest month and the driest month, and altitude were the main environmental factors affecting the distribution of *L. japonicus*. The priority protected areas of *L. japonicus* should be set in the southwest of central China.

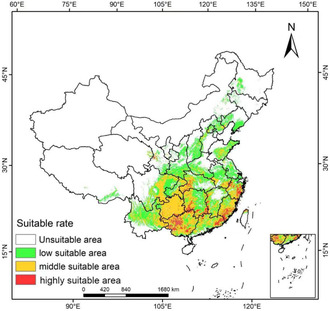

## 1 INTRODUCTION

In the sixth assessment report (AR6) of the Intergovernmental Panel on Climate Change (IPCC), it was pointed out that the impact and risk of climate warming are becoming increasingly complex, and this has produced a series of irreversible effects on ecosystems and human societies. Global warming will outweigh habitat destruction as the greatest threat to biodiversity in the coming decades (Li, Chang et al., 2021; Li, Tang et al., 2021). Climate change has a driving effect on the distribution of plants, and the suitable areas will range shift (Li, Chang et al., 2021; Li, Tang et al., 2021). The habitat of medicinal plants will be significantly reduced or severely migrated (Rana et al., 2020; Shen et al., 2021), posing a serious threat to the sustainable use of medicinal plants. Therefore, it is of great significance to study the response of plants to climate change for the protection (Gupta et al., 2021; Zhang et al., 2000, 2019).

In addition to climatic factors, soil is another important ecological factor affecting plant growth and distribution. There is a great contact surface between plant roots and soil, and there is frequent material exchange between plants and soil. The growth and development of plants require the soil to constantly supply certain water, nutrients, temperature, and air, and sufficient soil fertility is the basis for the normal growth and development of plants (Ma et al., 2020; Yuan et al., 2020). The effects of soil factors on plant growth and distribution are interrelated. The change in one factor can affect other factors and form a complex interaction network. Understanding soil factors and their effects on plants can help to select suitable plant species for specific soil conditions and implement soil management measures to optimize plant growth and productivity.

There are many models for predicting species distribution at home and abroad, such as ecological niche factor analysis (ENFA), bioclimatic analysis system (BIOCLIM), genetic algorithm for rule‐set production (GRAP), and maximum entropy mode (MaxEnt). Among them, the MaxEnt has higher modeling accuracy (Phillips & Miroslav, 2008; Zhu et al., 2022), and even in the absence of clear species distribution coordinates, better prediction results can be obtained by determining the latitude and longitude according to the existence points (Liu et al., 2018). It is considered to be one of the most effective prediction methods (Li, Chang et al., 2021; Li, Tang et al., 2021; Zhan et al., 2022). The MaxEnt can use the location information of species distribution and environmental variable data to fit the probability distribution with the largest entropy value to analyze and predict the potential geographical distribution pattern of species (Phillips & Miroslav, 2008). It can not only integrate multiple environmental variables when predicting potential distributions but also obtain the main influencing factors and adaptation range that affect the growth of the species. It has been widely used to predict the possible distribution of species and to study ecological characteristics (Zeng et al., 2021).

**FIGURE 1:**
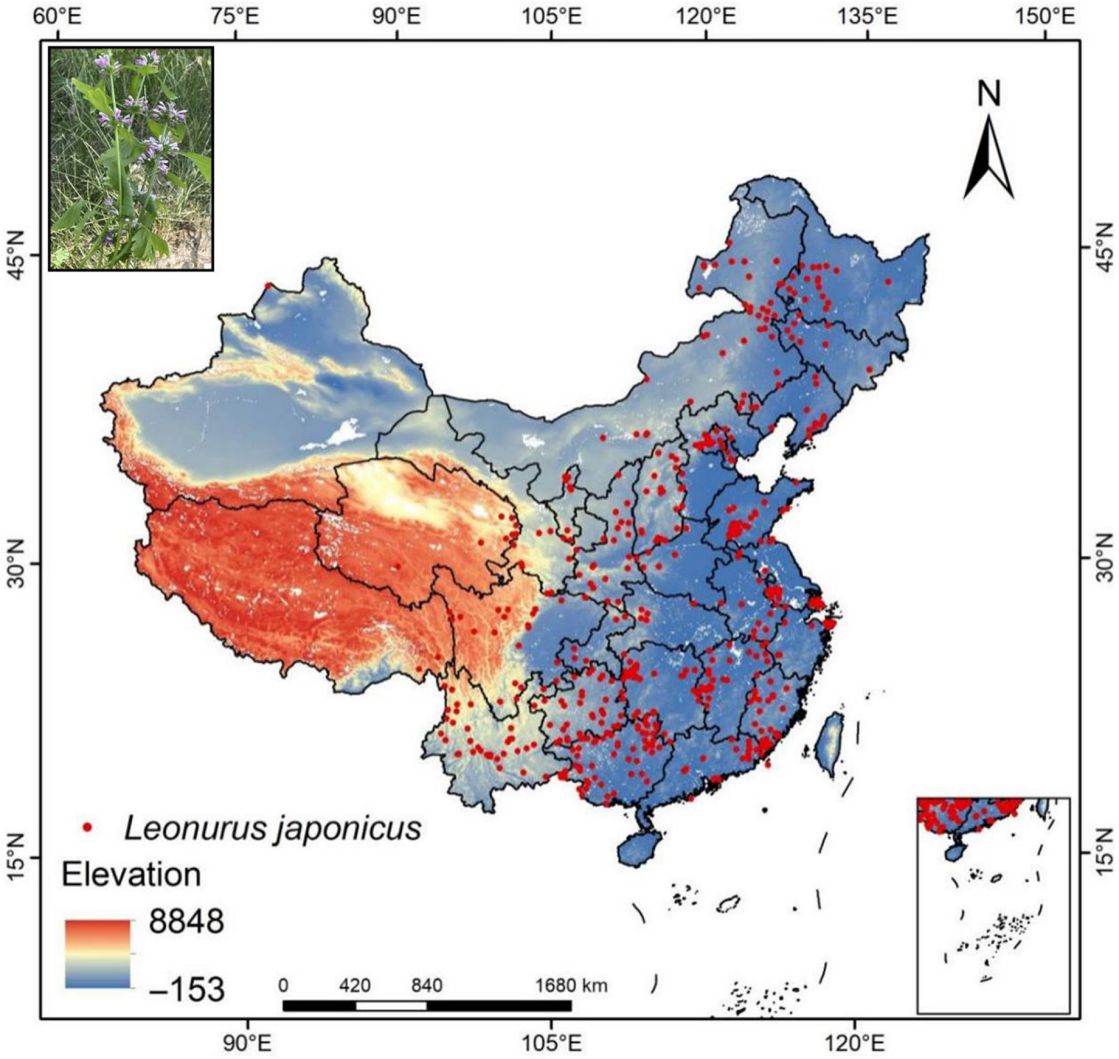
Geographical map of the distribution point of *Leonurus japonicus*.

*Leonurus japonicus* (on the top left of Figure 1) is a traditional Chinese medicinal plant. Its fresh or dry aboveground parts can be made into a medicine, which has the effects of regulating menstruation, diuresis, and swelling; removing blood stasis; promoting blood circulation and hemostasis; and preventing miscarriage (Wang et al., 2022; Zhang et al., 2000). It was used as a gynecological drug in traditional Chinese medicine during past dynasties, and the market demand is great (Jiang, 2009). The products have been widely used in health food (Zhang et al., 2014), cosmetics, and pharmaceutical industries and have broad market prospects (Li & Miao, 2023). In addition, *L. japonicus* plays an important role in the ecosystem. It can maintain the local ecological balance, reduce soil erosion, and improve water quality, which used for the construction of ecological landscape. With the exploitation and utilization, wild *L. japonicus* source is unable to meet the demand. Habitat protection and artificial planting are the basic ways to solve this problem (Jia et al., 2019).

At present, the researches on *L. japonicus* mainly focus on its pharmacology and chemical composition analysis, but the influencing factors affecting the suitability distribution of *L. japonicus* are not clear. With the change in climate, the research on the distribution and spatial pattern change in *L. japonicus* under the future climate change scenario has not been reported. Therefore, in order to scientifically clarify the distribution of *L. japonicus* and its response to future climate change, this study used the optimized MaxEnt model and ArcGIS V10.4.1 software to simulate and predict the current (1970–2020) and future 2050s (2041–2060) and 2090s (2081–2100) potential distribution of *L. japonicus* in China. This study focuses on three key objectives: (i) predicting the distribution pattern of the potentially suitable area of *L. japonicus* in China under current climate conditions and dividing them into different suitability grades; (ii) analyzing the relationship between the predicted potential distribution area of *L. japonicus* and the main environmental factors; and (iii) predicting and comparing the potentially suitable area and the change trends of *L. japonicus* under different climate conditions in the 2050s and 2090s. This study will provide a scientific basis for the development and utilization of *L. japonicus* resources.

## 2 MATERIALS AND METHODS

### 2.1 Collection of species geographical distribution data

By sorting out the information of *L. japonicus* specimens recorded in the Chinese Digital Herbarium (CVH, http://www.cvh.ac.cn/), and combining with the *L. japonicus* presence points in the Global Biodiversity Information Platform (GBIF, https://www.gbif.org/), the existing distribution positions of *L. japonicus* were preliminarily obtained, and then the corresponding latitude and longitude coordinates of each distribution point were obtained by Baidu coordinate system. The R software package “CoordinateCleaner” was used to removing records without coordinate precision and suspected outliers. Based on the “subset” “clean_coordinates” operation in CoordinateCleaner, we obtained the results of bias corrections on the datasets. We obtained a total of 904 existence points. In order to deal with the spatial autocorrelation of the occurrence points, we used the spThin package (Aiello‐Lammens et al., 2015) in R environment to delete the repeated points within a buffer of a 4.5‐km radius, which retained 489 occurrence points (Figure 1).

### 2.2 Selection and processing of environmental variables

Species’ ecological niches are affected by climate, topography, biology, and other factors. In consideration of the comprehensiveness and complexity of ecological factors, 34 environmental variables which could reflect species' ecological niches were selected. The list included 19 bioclimatic factors, 14 soil factors, and a topographic factor (altitude).

The current (1970–2000), 2050s (2041–2060), and 2090s (2081–2100) bioclimatic factor data used in this research were derived from the world climate database Worldclim (http://www.worldclim.Org), and the pixel size of the data was 2.5 arc‐minutes (−5 km). The climate data of the 2050s and 2090s were obtained from the Beijing Climate Center‐Climate System Model‐Medium Resolution (BCC‐CSM2‐MR), one of the Coupled Model Inter‐Comparison Project Phase 6 (CMIP6) datasets, which included three scenarios: sustainable development (SSP126), intermediate development (SSP245), and conventional development (SSP585). SSP scenarios have a high accuracy and separation rate and can integrate local development factors, and so are more convincing than CMIP5 data. The data of soil factors and topographic factors were obtained from the World Soil Database (HWSD) of the FAO (http://www.fao.org/faostat/en/#data), and the provincial national vector map was from China's Ministry of Natural Resources (http://www.mnr.gov.cn/).

In the application of species distribution models, we used variance inflation factor (VIF) to screen and Pearson correlation to reduce the multicollinearity between multiple environmental variables. At the beginning, we selected a total of 34 environmental factors, including bioclimate, soil, and terrain. On this basis, we considered the importance of variables obtained by the jackknife technique, using Pearson and VIF to check the correlation and importance of environmental factors. Pearson correlation analysis and multicollinear VIF analysis were performed with ENMeavl package (Muscarella et al., 2015) in R environment, and the environmental factors with correlations less than 0.7 and VIF values less than 5 were initially screened (Zhang et al., 2017). VIF variance expansion factor is also called the reciprocal of tolerance. When VIF < 5, there is no multicollinearity among factors; when 10 < VIF < 100, there is multicollinearity among factors; when 100 < VIF, there is serious interfactor multicollinearity. We use usdm (Naimi, 2015) package to calculate the VIF based on the above method, and 12 environmental factors that are relatively important to the geographical distribution of *L. japonicus* were screened out of 34 environmental factors (Table 1).

**TABLE 1:** Environmental aspects used in the modeling.

Type	Variable code	Environmental factor	Unit
Climatic factor	bio4	Temperature seasonality	1
	bio5	Max temperature of warmest month	×10°C
	bio6	Min temperature of coldest month	×10°C
	bio7	Temperature annual range	×10°C
	bio13	Precipitation of wettest month	mm
	bio14	Precipitation of driest month	mm
Topographic factors	elev	Altitude	m
Soil factor	t_cec_soil	Cation exchange capacity of topsoil	%
	t_ece	Electrical conductivity of topsoil	dS/m
	t_gravel	Gravel volume in topsoil	%
	t_oc	Organic carbon content in topsoil	%
	t_silt	Silt content in topsoil	%

### 2.3 Construction of the habitat suitability model

In this research, the MaxEnt model was used to predict the habitat suitability of *L. japonicus* in three periods. A total of 489 occurrence points and 12 environmental factors were imported into MaxEnt 3.4.4 software for modeling. We used 75% of the distribution samples randomly chosen as training data for modeling, and 25% of the distribution samples were used as test data to evaluate the model's predictive performance. Bootstrap replicates were set to 10, and the distribution value was output in the Cloglog form. The relative influence of each environmental factor was evaluated by the jackknife analysis method, and the most important variable factor was determined by combining the percentage contribution of each environmental factor and the replacement importance value.

The jackknife analysis method represents the importance of the explanatory variables based on the arrangement and reveals the importance of the variables. The accuracy value of the AUC‐ROC of the test data represents the model fitting degree and indicates the prediction reliability of the model (Phillips et al., 2006). The evaluation result of the AUC is in the range of 0.5–1.0. An AUC <0.6 suggests that the prediction result is unqualified; values between 0.6 and 0.7 indicate poor performance; values between 0.7 and 0.8 indicate general performance; values between 0.8 and 0.9 indicate good performance; values between 0.9 and 1.0 indicate excellent performance (Fielding & Bell, 1997).

### 2.4 Optimization of the model

Using machine learning algorithms, sample data are required to randomly or systematically sample the study area. In practice, the sample data obtained are biased toward more easily accessible areas in the species distribution space. In practice, the existence points are spatially biased toward areas that easier surveyed. Therefore, different sampling strategies result in uneven distribution of recorded data, inconsistent with the real spatial ecology of the target species (Shabani et al., 2016). This spatial bias leads to overrepresentation (spatial aggregation) of regions with high data density input in the model (Ahmadi et al., 2023), so machine learning algorithms are optimized through parametric modeling methods, such as fine‐tuning the MaxEnt model with the ENMeavl (Muscarella et al., 2015) package. Referring to Robert Muscarella's latest optimization method, the Checkerboard2 method was used to divide the study area into four bins. This masked geographic structure method can better adjust the model regularization level. The MaxEnt model regularization level consists of two parameters, the regularization multiplication value (RM) and the feature combination (FC) optimized by calling the ENMeval packet in the R environment. The MaxEnt model provides five features: a linear feature (L), a quadratic feature (Q), a fragmented feature (H), a multiplicative feature (P), and a threshold feature (T). In this research, the adjusted parameters of MaxEnt software were RM = 1.5 and FC = H; to optimize the MaxEnt model, the RM was set to 0.5–4. Each increase was by 0.5, and there were a total of eight control frequency doublings. Meanwhile, six combinations with one or more features were used: L; L and Q; H; L, Q and H; L, Q, H and P; and L, Q, H, P and T. According to the permutations and combinations, 48 parameter combinations were calculated. The ENMeval package uses the above 48 combinations of parameters to test the complexity of the model based on the delta AICc value and a 10% test miss rate, where the lower the value, the more accurate the model prediction (Steven et al., 2017; Zhao, Cui, Sun et al., 2021; Zhao & Fan, 2021).

### 2.5 Data processing

ArcGis10.4.1 software was used to classify and visualize the suitability of *L. japonicus*. The habitat suitability threshold of *L. japonicus* was predicted based on the MaxEnt model. The natural breakpoint method was used to classify the habitat suitability index of *L. japonicus*. The minimum level of threshold establishment was 0.47, and the distribution below this level was excluded (Liu et al., 2005, 2013). Therefore, the habitat suitability grade of *L. japonicus* was divided into unsuitable area (0–0.47), low suitable area (0.47–0.55), moderate suitable area (0.55–0.65), and most suitable area (0.65–1).

We fitted species distribution model based on the current time and then project the fitted model to future climatic scenarios. We added terrain and soil data to the future prediction, assuming that there is no change in soil terrain data within 100 years (Zhang et al., 2018). We compared the differences in the suitable areas of *L. japonicus* in different periods to obtain the change map of the spatial distribution pattern of *L. japonicus* under future climate change scenarios. The SDMTool (Naimi & Araújo, 2016) package in the R environment was used to calculate the centroid position of the suitable area of *L. japonicus* in the current and future periods, and the direction of range shift in suitable habitats of *L. japonicus* was reflected by the change in the centroid position. The geosphere (https://cran.r‐project.org/package=geosphere) package in the R environment was used to calculate the centroid range shift distance of *L. japonicus* under different climate change scenarios. The ArcGis overlay tool was used to overlay the current and future suitable area distribution data layers. The tool can merge different raster data into one output to define the specified set, reclassify the new layer, and divide the suitable level to obtain the final suitable zoning map of *L. japonicus*. In this study, three suitable classes of low suitable area, medium suitable area, and highly suitable area were taken as the total suitable area; the values of dominant environmental variables and the suitability of different periods were extracted from 489 distribution points to analyze the relationship between environmental factors and potential distribution areas.

### 2.6 Changes in the spatial pattern of the suitable distribution area for *L. japonicus*

Referring to Ye's et al. (2020) method, the potential geographical distribution results of *L. japonicus* in each period were compared with the current geographical distribution results, and the suitable distribution area of *L. japonicus* was retained, added, and lost. The spatial unit with a probability of species existence ≥0.47 was the suitable area for *L. japonicus*, and the spatial unit with a probability of species existence <0.47 was the unsuitable area (Zhao, Cui, Sun et al., 2021; Zhao, Cui, Wang et al., 2021; Zhao & Fan, 2021). On this basis, the existence/nonexistence (0,1) matrix of potential geographic distribution of *L. japonicus* under current and future climate change scenarios was established. The appropriate region is assigned a value (1) indicating the existence, while the inappropriate region is assigned a value (0) indicating the nonexistence. Based on the matrix tables 0,1, the changes in the spatial pattern of *L. japonicus* suitable distribution areas under current and future climate scenarios were further analyzed. Four types of suitable area changes were identified: new suitable area, lost suitable area, retained suitable area, and unsuitable area. Based on the current suitable distribution area of *L. japonicus*, the area changes in the current and future suitable distribution areas of *L. japonicus* were calculated. In the context of current and future climate change, changes in the spatial pattern of potential suitable areas are defined as follows: matrix value 0 → 1 represents new suitable areas, 1 → 0 represents lost suitable areas, 1 → 1 represents reserved suitable areas, and 0 → 0 represents unsuitable areas (Li et al., 2019; Ye et al., 2020).

## 3 RESULTS

### 3.1 Model optimization and accuracy evaluation

Based on 489 distribution points, 12 layers of environmental variables, and the AIC information criterion, the MaxEnt model was used to simulate and predict the potential distribution area of *L. japonicus*. Running MaxEnt, its default parameter settings are RM = 1, FC = LQHPT, and delta.AICc = 6.99. The results show that when RM = 1.5, FC = H, delta.AICc = 0, the model is optimal. And the 10% training omission rate value is lower than the default parameter model (Table 2), which is 13.48% lower than the default value.

**TABLE 2:** Evaluation results of the MaxEnt model under different parameter settings.

Model evaluation	Feature combination	Regularization multiplier	Value of delta Akaike information criterion corrected	10% training omission rate
Default	LQHPT	1	6.99	0.29873
Optimized	H	1.5	0	0.25847

**FIGURE 2:**
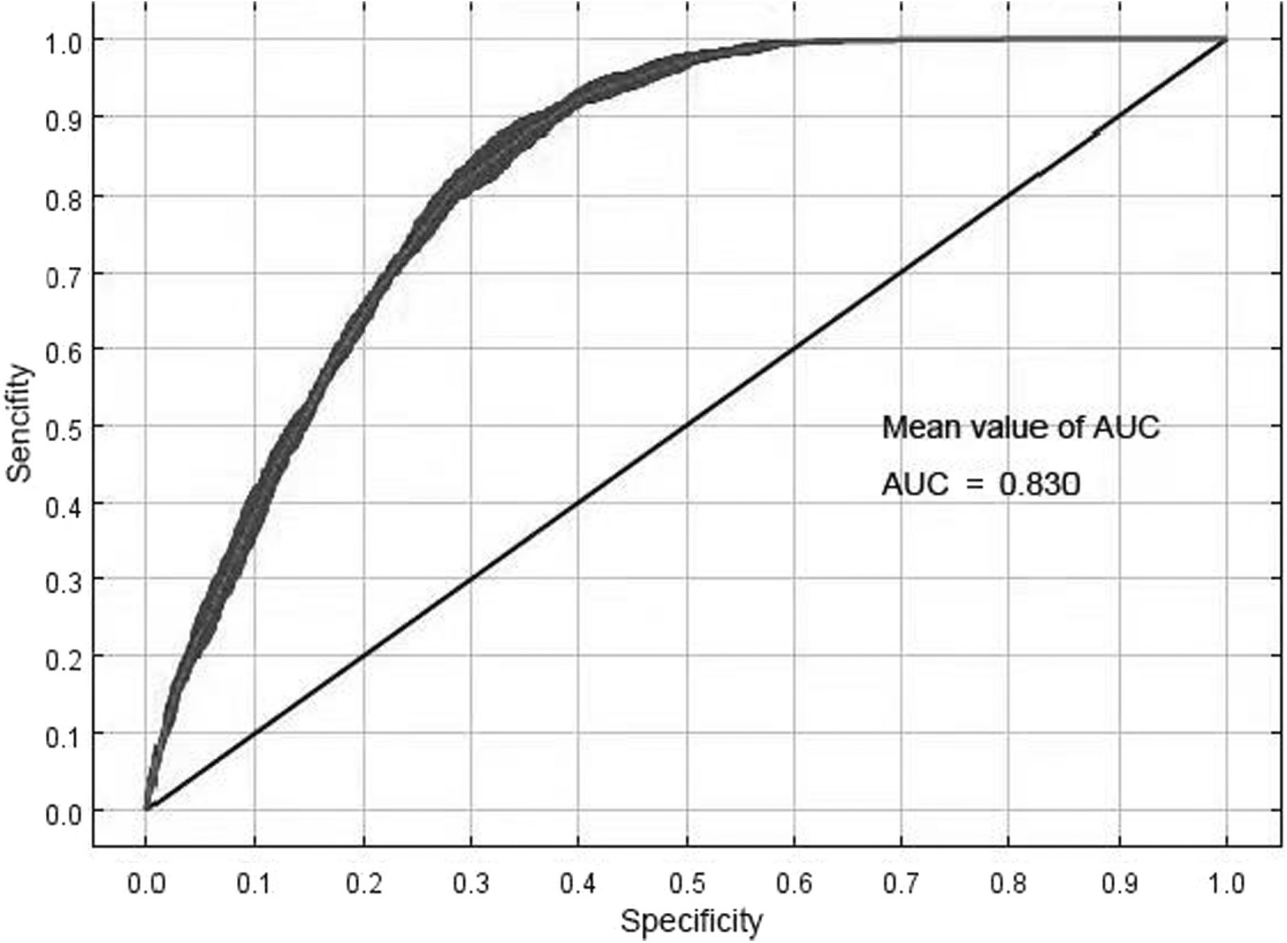
ROC response curve under the MaxEnt model.

Therefore, the control frequency doubling RM = 1.5 and the feature combination FC = H were selected as the final parameters of the model. The AUC value of the simulation training under these parameters was 0.830. The standard deviation was 0.006 (Figure 2), indicating that the prediction results were accurate.

### 3.2 Potential geographical distribution of *L. japonicus* in China

**FIGURE 3:**
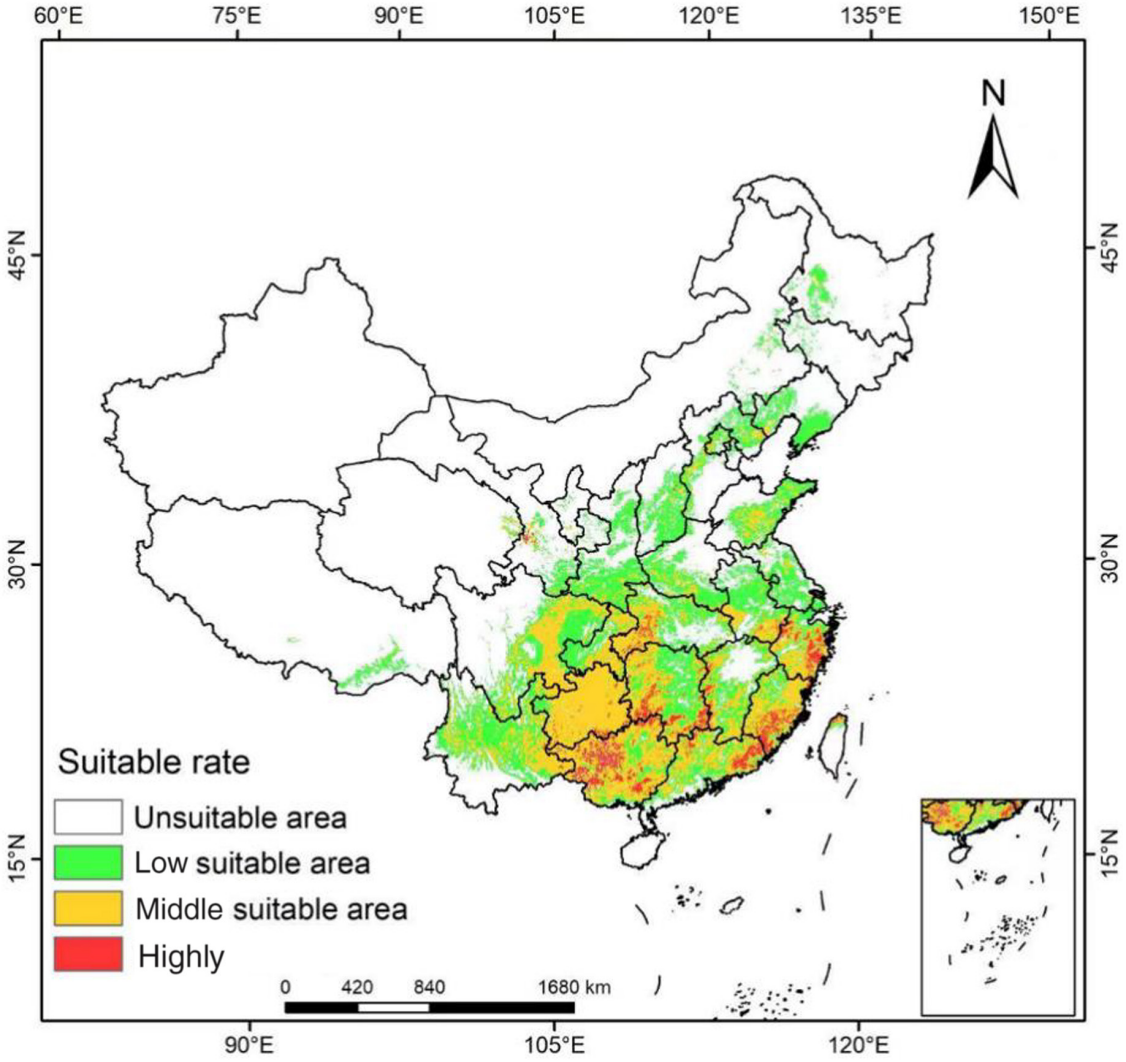
Suitable distribution of *Leonurus japonicus* in China via MaxEnt.

The MaxEnt model was used to simulate the distribution map of the suitable area of *L. japonicus* in the current period (Figure 3). The area of highly suitable, moderately suitable, and low suitable habitats of *L. japonicas* for the current time was 135,620, 1,043,933, and 1,202,780 km^2^, respectively. These areas were distributed in 27 provinces and cities: Shanxi, Shaanxi, Shandong, Henan, Jiangsu, Zhejiang, Anhui, Hubei, Chongqing, Sichuan, Yunnan, Guizhou, Jiangxi, Fujian, Guangxi, Guangdong, and other places. Highly suitable areas were concentrated in southwestern Hubei, western Guangxi, southeastern and northwestern Zhejiang, northeastern Guangdong, southern Fujian, eastern Hunan, and southwestern Jiangxi.

### 3.3 Prediction of suitable growing areas for *L. japonicus* under future climate scenarios

Under the future climate scenarios, the predicted total suitable area of *L. japonicus* in the 2050s and 2090s would be reduced by varying degrees compared with the current period (Table 3). Compared with the current period, in 2050s the total suitable area of *L. japonicus* is projected to decrease by 47.97%, 49.93%, and 47.73% under the SSP126, SSP245, and SSP585 scenarios, respectively. Compared with the 2050s, the total suitable area in the 2090s showed a downward trend under SSP245 and SSP585 scenarios, with decreases of 20.54% and 54.38%, respectively, while increasing significantly under the SSP126 scenario to 5.98 × 10^4^ km^2^, 5.80% greater than that of 2050 time‐period scenarios in the future ×10^4^ Km^2^.

**TABLE 3:** Suitable growing area of *Leonurus japonicus* in China, under different climate.

Climate change scenarios	Unsuitable area	Low‐grade suitable area	Moderately suitable area	Highly suitable area	Total suitable area
Current	762.07	119.99	64.27	13.67	197.93
SSP126‐2050s	857.02	71.66	26.71	4.61	102.98
SSP126‐2090s	851.04	71.66	32.36	2.18	108.96
SSP245‐2050s	860.89	72.84	24.80	1.47	99.11
SSP245‐2090s	881.25	60.57	17.69	0.50	78.75
SSP585‐2050s	856.54	74.31	25.87	3.28	103.46
SSP585‐2090s	912.80	34.28	12.31	0.61	47.20

**FIGURE 4:**
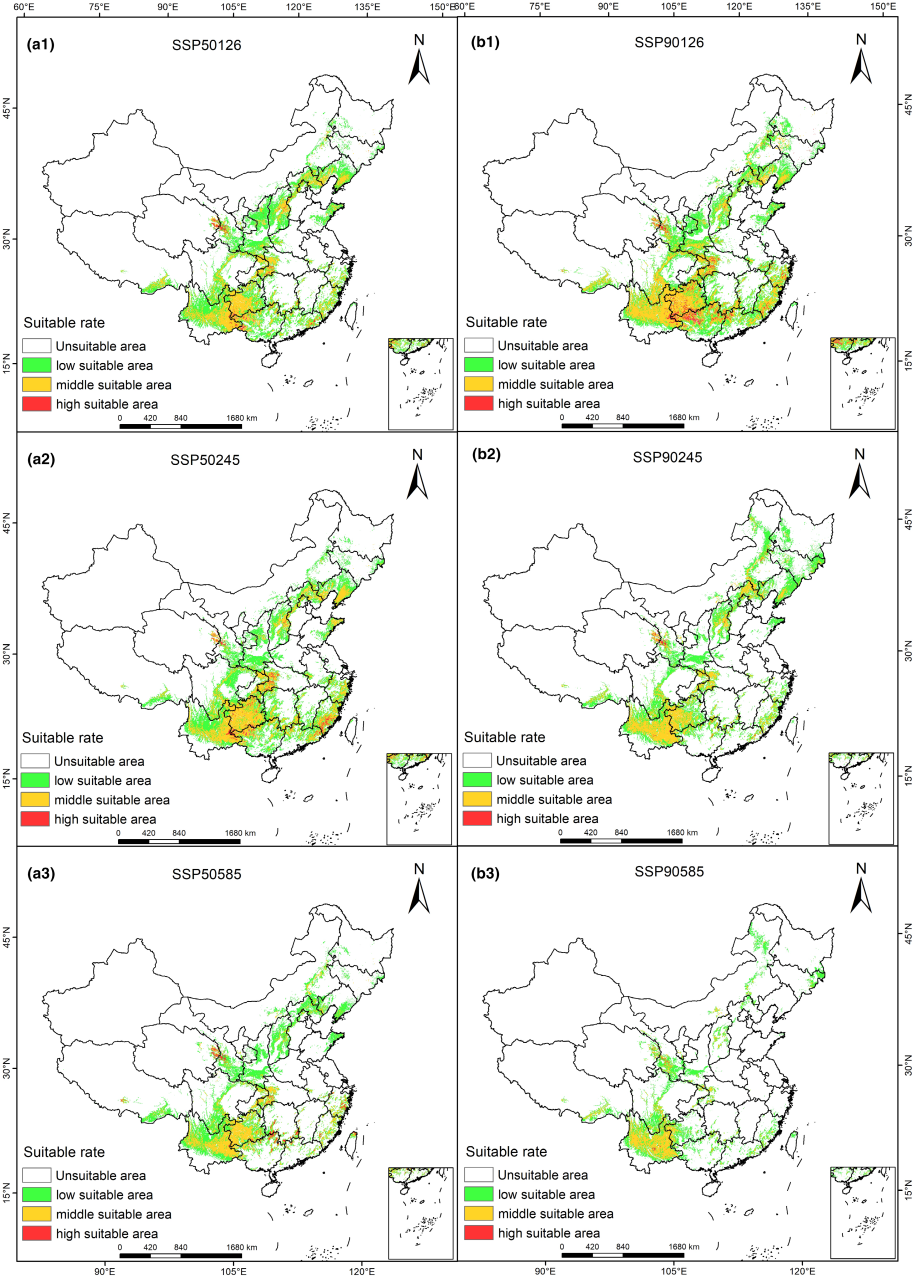
Suitable habitat distribution for *Leonurus japonicus* in China in the future under different climate change scenarios.

The response of *L. japonicus* to climate change differs across the projected suitable areas for the 2050s and 2090s. Figure 4 shows that there are variations in the trends. Under the SSP585 scenario, the highly suitable area for *L. japonicus* is most impacted by climate change. By the 2050s, the highly suitable area decreases by 10.39 × 10^4^ km^2^, which is 76.00% lower than the current area. By the 2090s, this reduction extends to 13.06 × 10^4^ km^2^, indicating a decrease of 95.55% compared with the current area. Under the SSP126, the highly suitable area for *L. japonicus* in the 2090s experiences the smallest reduction. There is a decrease of 11.49 × 10^4^ km^2^, which is 84.02% of the current area. In the SSP245 scenario, the highly suitable area for *L. japonicus* in the 2050s shows a decrease of 12.21 × 10^4^ km^2^, accounting for an 89.28% reduction. However, by the 2090s, the reduction compared with the 2050s is 0.97 × 10^4^ km^2^, indicating a decrease of 66.18%. Across all three climate scenarios, the suitable area of *L. japonicus* exhibits a decreasing trend, with the species being most sensitive to climate change in the SSP245 and SSP585 scenarios.

**FIGURE 5:**
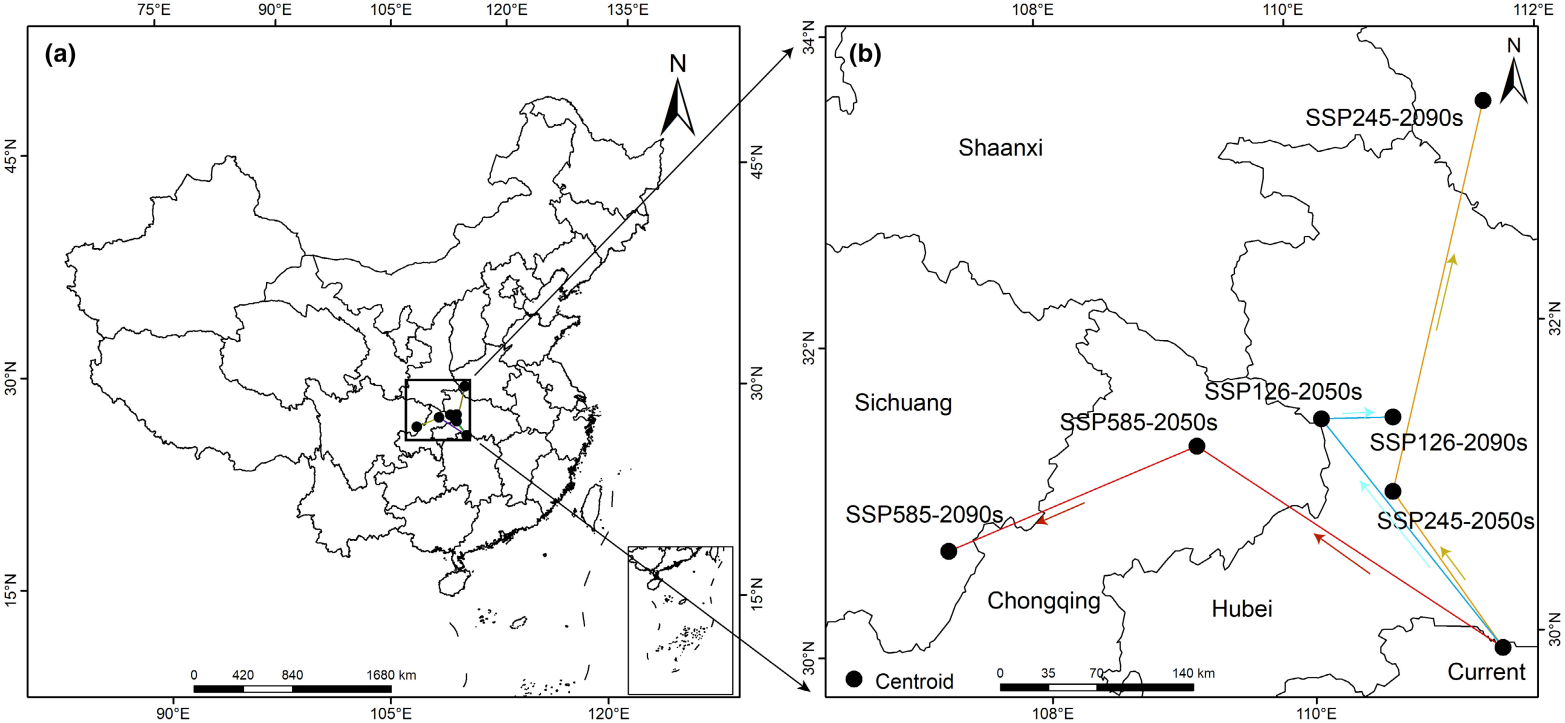
Geographical distribution changes in the centroid of the suitable growing area of *Leonurus japonicus* under different climate change scenarios.

In terms of spatial pattern, there were large differences in suitable range shift locations of *L. japonicus* under different climate scenarios, but the overall migration trend was more consistent; the overall trend migrates northward (Figure 5). At present, the centroid of the suitable area of *L. japonicus* is in Lixian County, Changde City, Hunan Province (111.43°E, 29.90°N). Under the climate scenario SSP245 in the 2090s, the center of mass of the suitable area of *L. japonicus* would be the farthest north. At this time, the center of mass of the suitable area of *L. japonicus* would be located in Xixia County, Nanyang City, Henan Province (111.55°E, 33.43°N); the migration distance would be 391,314 m. Under the climate scenario SSP585‐2090s, the movement distance of the centroid of the suitable area of *L. japonicus* to the northwest is 413,003 m, moving to Dazhu County, Dazhou City, Sichuan Province (107.23°E, 30.68°N). Under future climate change scenarios, global warming and humidification will cause the mass center of the suitable growing area of *L. japonicus* in China to move northward as a whole, and the migration location would trend toward further northwestward expansion.

### 3.4 Evaluation of environmental factors

The results of the environmental factor evaluation are shown in Table 4. The factors contributing more than 5% to the potential geographical distribution of *L. japonicus* (Zhan et al., 2022) were the minimum temperature of coldest month(12.2%), the precipitation of wettest month (53.8%), the precipitation of driest month (5.7%), and altitude (20.9%). The total contribution rate of these four environmental factors was as high as 92.6%. The factors with more than 5% of the importance value were the maximum temperature of warmest month (10.6%), the minimum temperature of coldest month (19.4%), the precipitation of wettest month (40.3%), the precipitation of driest month (5.8%), and the altitude (11%). The importance values of these five environmental factors were as high as 87.1%. Considering these five environmental factors as the main environmental factors, precipitation was the dominant factor.

**TABLE 4:** Contribution rate and importance of environmental variables.

Environmental variable	Contribution (%)	Importance (%)
Temperature seasonality	2.3	4.2
Max temperature of warmest month	1.3	10.6
Min temperature of coldest month	12.2	19.4
Temperature annual range	0.2	1.7
Precipitation of wettest month	53.8	40.3
Precipitation of driest month	5.7	5.8
Altitude	20.9	11
Cation exchange capacity of topsoil	0.5	1.4
Electrical conductivity of topsoil	1.3	2.3
Gravel volume in topsoil	0.5	0.6
Organic carbon content in topsoil	0.8	1
Silt content in topsoil	0.7	1.7

### 3.5 Dynamic changes in the suitable habitats for *L. japonicus* under different combinations of climate scenarios

**FIGURE 6:**
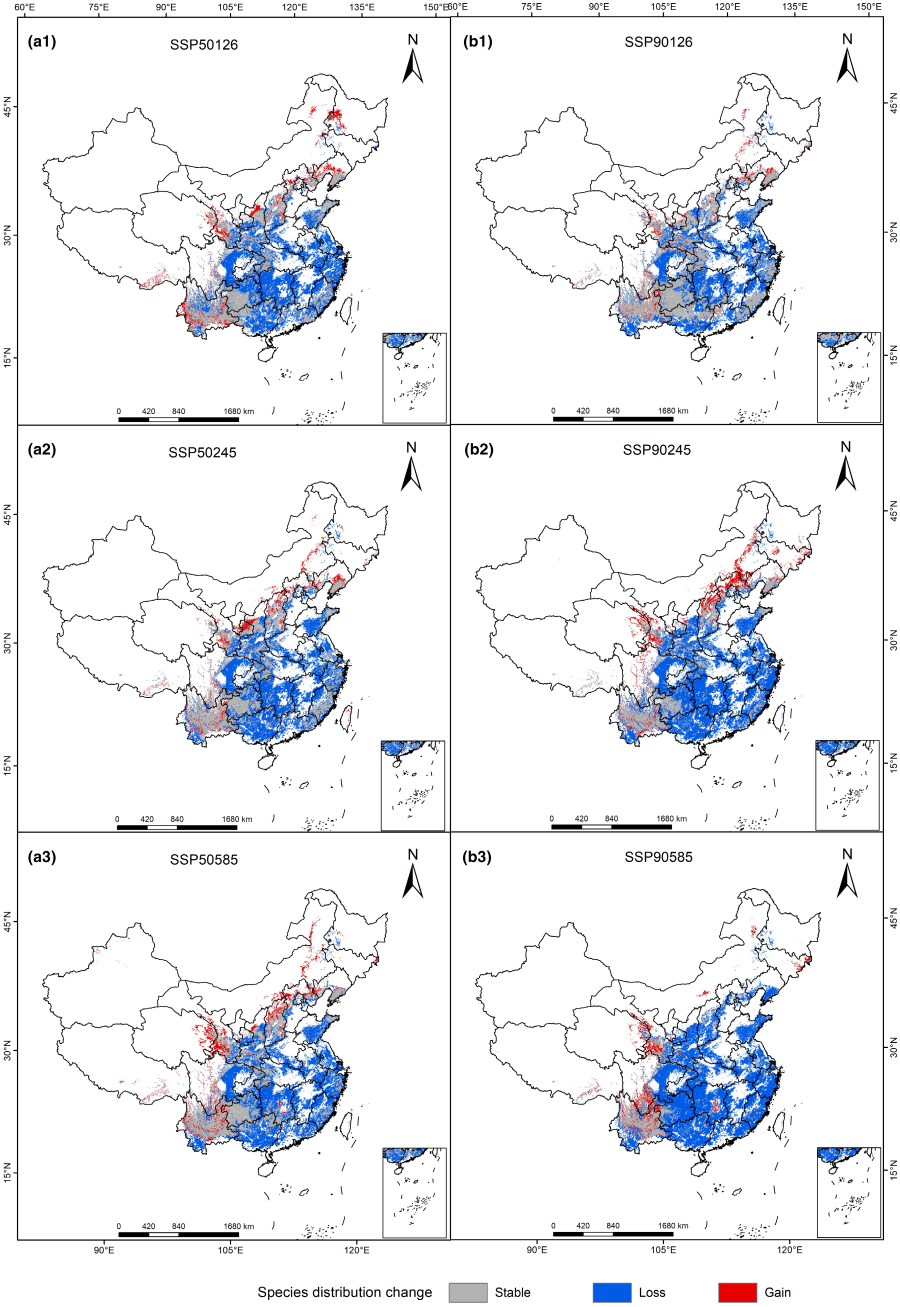
Spatial changes in *Leonurus japonicus* in China in the future under different climate change scenarios.

The spatial pattern changes in *L. japonicus* suitable areas under several climate scenarios in the future were compared and analyzed (Figure 6). The results showed that the suitable habitats of *L. japonicus* in different climate scenarios experienced different degrees of shrinkage in the future climate change (Table 5). In the next three climate scenarios, only a small part of the current suitable area of *L. japonicus* is retained. The reserved area ranges from 31.42 to 97.71 × 10^4^ km^2^, and the retention rate is between 15.87% and 49.37%. In the next three climate scenarios of *L. japonicus*, the increase area of *L. japonicus* ranged from 11.25 to 24.57 × 10^4^ km^2^, with an increase rate of 5.68%–12.41%. The increase area of *L. japonicus* mainly appeared in the north and west of the current suitable area. The loss area of *L. japonicus* ranged from 100.22 × 10^4^ to 166.51 × 10^4^ km^2^, and the loss rate ranged from 50.63% to 84.13%. The loss area of *L. japonicus* mainly appeared in the south of the current suitable area of *L. japonicus*. Through the comparative analysis of the spatial pattern changes in the potential habitats of *L. japonicus* under different climate change scenarios in the future, it shows that the suitable habitats of *L. japonicus* change with climate change and its response to climate change is generally consistent. The regional change rate is the lowest in the SSP126‐2090s scenario and the highest in the SSP585‐2090s scenario.

**TABLE 5:** Changes in the distribution area of *Leonurus japonicus* in different periods under different scenarios.

Period	Climate scenario	Habitat area (×10^4^ km^2^)	Loss (×10^4^ km^2^)	Stable (×10^4^ km^2^)	Gain (×10^4^ km^2^)	Species range change (%)	Percentage loss (%)	Percentage gain (%)
Current		197.93						
2041–2060	SSP126	102.98	114.56	83.37	19.61	−47.972	57.88	9.91
	SSP245	99.11	115.30	82.63	16.48	−49.929	58.25	8.33
	SSP585	103.46	118.15	79.78	23.68	−47.728	59.69	11.96
2081–2100	SSP126	108.96	100.22	97.71	11.25	−44.95	50.63	5.68
	SSP245	78.75	143.75	54.18	24.57	−60.213	72.63	12.41
	SSP585	47.20	166.51	31.42	15.78	−76.156	84.13	7.97

## 4 DISCUSSION

### 4.1 Optimization of the model for predicting the current suitable area of *L. japonicus*

This study used the ENMeval package to optimize the model based on climate, topography, and soil factors. This method limits the background data to the area corresponding to the calibration location, so that the potential geographical distribution area simulated by MaxEnt covers the current distribution points. This method allows the adjustment of model parameters to improve the performance of the MaxEnt model, and then an adjustment test is performed by changing the regularization level, thereby reducing the complexity of the model. Finally, the accuracy of the model is measured by improving the fitting degree between the predicted results and the actual distribution area and by visual inspection of the geographic prediction map (Steven et al., 2017). The MaxEnt model with optimized parameters can efficiently reduce the complexity of the model and improve the fitting degree between the predicted results and the actual results, and it predicts the distribution of species well. The response curve becomes smooth and approaches the normal distribution curve, conforming to the Shelford tolerance rule (Li et al., 2018; Ouyang et al., 2019; Steven et al., 2017).

*Leonurus japonicus* grows in various habitats, mostly in areas with the sun exposure, and it can grow in general soils. It is distributed in Anhui, Fujian, Guangdong, Guangxi, Guizhou, Hainan, Hebei, Heilongjiang, Henan, Hubei, Hunan, Jiangsu, Jiangxi, Jilin, Liaoning, Inner Mongolia, Shaanxi, Shandong, Shanxi, Sichuan, Yunnan, Zhejiang, and other places (http://www.iplant.cn/info/Leonurus%20japonicus?t=f). In this research, based on the optimized maximum entropy model, the current suitable areas of *L. japonicus* were predicted, and the results were consistent with the actual distribution, indicating that the MaxEnt model was reliable and accurate for predicting the distribution of *L. japonicus*.

### 4.2 Restriction of environmental variables on geographical distribution of *L. japonicus*

The maximum temperature of warmest month, the minimum temperature of coldest month, the precipitation of wettest month, altitude, and the precipitation of driest month were the main environmental factors affecting the distribution of *L. japonicus*. To understand the influence of main environmental factors on the distribution of *L. japonicus*, the relationship between distribution probability and ecological factors was judged according to the response curve. It was assumed that when the distribution probability of *L. japonicus* was greater than 0.5, the corresponding environmental factors were suitable for plant growth (Guo et al., 2017; Jia et al., 2019). In this study, when the probability was greater than 0.5, the highest temperature range of the hottest month was 26.8–33°C, and the highest temperature of the hottest month most suitable for the growth of *L. japonicus* was about 31°C. When the probability was greater than 0.5, the range of the minimum temperature of coldest month was −8 to 11.5°C; the minimum temperature of coldest month that was most suitable for the growth of *L. japonicus* was about 8.3°C, a result that was consistent with the research of Wang and colleagues (Wang & Yin, 2015) suggesting that the suitable temperature for the growth of *L. japonicus* is 22–30°C, at high temperatures above 35°C the plant grows well, and the growth is slow below 15°C. In this forecast, when the probability was greater than 0.5, the wettest monthly precipitation was in the range of 178–370 mm, and the wettest monthly precipitation that was most suitable for the growth of *L. japonicus* was about 240 mm. The most suitable altitude for the growth of *L. japonicus* was about 200 m, which fully reflects the warm and humid climate preference of *L. japonicus* and is suitable for growth below a 1000 m altitude (Wang & Yin, 2015).

This study shows that precipitation, topography, and temperature determine the potential geographical distribution of *L. japonicus* on a certain scale. Although precipitation plays an important role in the potential distribution pattern of *L. japonicus*, topography and temperature factors will also redistribute precipitation resources to a certain extent. Therefore, the effects of temperature, precipitation, and topography on the potential distribution pattern of *L. japonicus* cannot be ignored. The interactions between these factors affect the potential distribution pattern of *L. japonicus*.

### 4.3 Effect of climate warming on geographical distribution of *L. japonicus*

Under the three shared socioeconomic pathways (SSPs) scenarios in the future, high humidity and temperature will expand the distribution range of the suitable area of *L. japonicus* to the north. Compared with the current suitable area, the 2050s shows a significant decrease, which is significantly greater than the decrease of 2090s. The response to climate change was most sensitive under the SSP245 and SSP585 scenarios. Under the SSP126 scenario, the distribution of suitable areas for *L. japonicus* increased significantly, indicating that sustainable development has an important impact on biological growth, reproduction, and the improvement of the ecological environment. Under different economic path change scenarios in the future, the response of the spatial pattern of the suitable area of *L. japonicus* to the change is generally consistent. With the increase in climate warming, the overall migration of the spatial location of the suitable area of *L. japonicus* becomes greater. When the shared socioeconomic path is the same, except for the geometric center point of the 2090s suitable area under the SSP585 scenario, under the low‐moderate socioeconomic paths, the geometric center of the 2090s *L. japonicus* suitable area shows a trend of northward migration compared with the 2050s, and the migration distance is the largest under the SSP245‐2090s scenario. The range shift trend of *L. japonicus* is consistent with the range shift of species to high latitudes under the background of global warming (Liu et al., 2022).

### 4.4 Response of the spatial distribution pattern of *L. japonicus* to climate change in China

This study explained the changes in the geographical distribution of suitable habitats of *L. japonicus* from the aspects of suitable habitat expansion and loss. Under the future climate change scenario, the overall trend of suitable habitat for *L. japonicus* is consistent; that is, it shows a downward trend. The loss rate of suitable area of *L. japonicus* is much greater than the increase rate. This result is consistent with the results of Yu's et al. (2019). Under the climate scenarios of SSP245‐2090s and SSP585‐2090s, the total suitable habitat of *L. japonicus* decreased significantly, and the spatial variation range of suitable distribution area was larger, which further indicated that the distribution of *L. japonicus* was greatly affected by climate change and sensitive to climate change. This result is consistent with the research conclusions of *Phoebe chekiangensis*, *Lonicera oblata*, *Eleutharrhena macrocarpa*, and *Phoebe bournei* (Wu et al., 2016, 2021; Ye et al., 2021; Zhao, Cui, Sun et al., 2021; Zhao, Cui, Wang et al., 2021; Zhao & Fan, 2021). The change in geographical distribution is more significant in the border areas of suitable habitats. The increase in suitable habitats is located on the boundary of suitable areas and is a sensitive area for species to respond to climate change (Diamond et al., 2011; Thuiller et al., 2005). The increase area of *L. japonicus* in the future period is located in the north of the current suitable habitat, and the loss area is located in the south of the current suitable habitat. It indicates that the suitable habitat of *L. japonicus* will generally migrate to areas with high latitude in the future. The results of this study also verify that the migration direction of the centroid of *L. japonicus* in the future climate scenario is correct, which is consistent with other research results that indicate climate warming leads to species migration to higher latitudes (Chen et al., 2011; Li et al., 2019; Ye et al., 2020).

### 4.5 Potential limitations

The main limitations of this study are summarized as follows. First, this study aims to predict the potential distribution area of *L. japonicus* in China. We only collected the data of distribution points in China, so the response curve of environmental factors cannot fully reflect the response of *L. japonicus* to the environment, especially in its global distribution area. Despite the above deficiencies, we used the optimized MaxEnt to obtain the predicted potential suitable area for *L. japonicus*. In China, it has high reliability. Second, this study only predicted the effects of climate, soil, topography, and other factors on *L. japonicus*, without considering the effects of bioecological factors such as species interaction and human activities (Ahmad et al., 2019). Therefore, the predicted potential suitable areas will deviate from the actual suitable areas (Zhu et al., 2022). Finally, in this study, the land‐cover conditions along with climate variables were used as input to the model; however, we assume that the land‐cover situation will remain unchanged in the future. Climatic factors are considered to be the main factors in the study of species distribution in other countries around the world. In order to better understand the impact of climate change on species distribution, intraspecific interactions and changes in land cover should be considered. With the continuous improvement of future information, how to introduce as many relevant variables as possible in computer model simulation is a problem worthy of further study. In future studies, we will adjust the distribution pattern according to the above deficiencies to guide *L. japonicus* more accurately.

## 5 CONCLUSIONS

In summary, this is the first study to use the optimized MaxEnt model to assess the impact of future climate change on the distribution of *L. japonicus*. The geographical constraints that may face the development of *L. japonicus* industry under climate change conditions were quantitatively evaluated. The results show that compared with the current climatic conditions, the suitable areas of *L. japonicus* will decrease to different degrees in all scenarios in the future, and the suitable areas of *L. japonicus* will shift to high latitudes with the increase in carbon emissions. This study can provide an adaptive management strategy for the planning of *L. japonicus* production areas in the future.

## AUTHOR CONTRIBUTIONS

**Yongji Wang:** Conceptualization (lead); writing – original draft (equal); writing – review and editing (equal). **Liyuan Xie:** Data curation (equal); formal analysis (equal); writing – original draft (equal). **Xueyong Zhou:** Methodology (equal); resources (equal); supervision (equal). **Renfei Chen:** Data curation (equal); funding acquisition (equal); software (equal); supervision (equal). **Guanghua Zhao:** Formal analysis (equal); project administration (equal); software (equal); validation (equal); visualization (equal). **Fenguo Zhang:** Funding acquisition (equal); investigation (equal); project administration (equal); supervision (equal); writing – review and editing (equal).

## FUNDING INFORMATION

This work was supported by the National Natural Science Foundation of China (grant nos. 41801027, 31700434, and 32101235), the Fundamental Research Program of Shanxi Province (grant nos. 202303021211250 and 202303021211252), and the Fund Program for the Scientific Activities of Selected Returned Overseas Professionals in Shanxi Province (grant nos. 20230025 and 20230027), the Research Project Supported by Shanxi Scholarship Council of China (2023–110), and the Science and Technology Innovation Project of Colleges and Universities in Shanxi Province (2021L274).

## CONFLICT OF INTEREST STATEMENT

The authors declare that they have no known competing financial interests or personal relationships that could have influenced the work reported in this paper.

## Data Availability

All data and analysis code will be made publicly available upon manuscript acceptance at Dryad (https://doi.org/10.5061/dryad.jdfn2z3gx).
